# Methane plume detection after the 2022 Nord Stream pipeline explosion in the Baltic Sea

**DOI:** 10.1038/s41598-024-63449-2

**Published:** 2024-06-19

**Authors:** Katarina Abrahamsson, Ellen Damm, Göran Björk, Carina Bunse, Amanda Sellmaier, Göran Broström, Volkmar Assmann, Adela Dumitrascu, Adele Maciute, Niten Olofsson, Milad Pourdanandeh

**Affiliations:** 1 Department of Marine Sciences, University of Gothenburg, Medicinaregatan 7b, 413 90 Gothenburg, Sweden; 2 Alfred Wegener Institute Helmholtz Centre for Polar and Marine Research, Telegrafenberg A45, 14473 Potsdam, Germany

**Keywords:** Biogeochemistry, Environmental sciences

## Abstract

On September 26th, 2022, the detonations at the gas pipelines Nord Stream 1 and 2 resulted in some of the largest non-natural releases of methane known. The distribution of methane in the surrounding seawater and the possible effects were not apparent. To trace the pathways of methane we recorded CH_4_ concentrations and the isotopic signal (δ^13^C-CH_4_) in seawater, and air. A week post-explosion, we detected methane concentrations up to 4 orders of magnitude above the natural Baltic Sea background. The released fossil methane created a distinct plume with δ^13^C-CH_4_ ratios differing from natural background values. The strong water stratification preserved the distribution pattern initiated by the explosion, shown by the laterally strong concentration gradient within the plume. Our analysis encompasses three stages of the explosion's impact; the initial sea-air methane release, measurements taken during our research expedition one week later, and a third stage triggered by the shift from summer to winter conditions as an outlook on how winter mixing and microbial activity will influence the plume.

## Introduction

Nord Stream is an infrastructure that comprises a network of offshore pipelines (Nord Stream 1 and 2) to provide Northern Europe with fossil gas. The pipeline systems run on the sea floor of the Baltic Sea from Russia to Northern Germany. The annual amount of gas that was transported was given as 60 × 10^12^ m^3^, which corresponds to approximately 20 × 10^12^ metric tonnes^1^. On the 26th of September 2022 the gas pipelines Nord Stream 1 and 2 abruptly, and simultaneously, started to leak at four locations in Swedish and Danish economic zones. Detonations were registered by several Danish and Swedish seismic stations and the pipeline leaks were associated with the explosions by the Swedish Coastguard.

The increased usage of submerged pipelines on a global scale has initiated a number of model studies of the release of gas into sea water from damages such as corrosion, but previous studies have primarily focused on smaller leakage sites^2^. The scale of the Nord Stream incident is among the largest known. There is only a limited number of scientific descriptions of similar sub-surface mega gas bubble events. For instance, the blow-out accident at site 22/4b in the North Sea has been extensively investigated regarding the intense release of methane and plume formation^3–5^. As methane is a more potent greenhouse compared to C0_2_, such release events add to the growing concern about the effects of anthropogenic climate change. Environmental implications on the marine environment include local impacts on water column carbon budgets and changes to the composition of microbial organisms that can tolerate and/or degrade hydrocarbons^6,7^. Subsequently, carbon from the methane plume could be recycled through the microbial food web^6^.

The scope of this paper is to estimate the amount of methane dissolved in seawater in relation to budget estimates of fossil methane directly released to the atmosphere after the explosion. Further, using the δ^13^C-CH_4_ ratios this study differentiates between natural background methane and pipeline-released methane in seawater. Recently produced methane in the Baltic Sea results from microbial degradation of organic matter, while within the Nord Stream pipelines, fossil methane from Siberian gas reservoirs was transported. Compared to the δ^13^C-CH4 ratios of background methane ranging between − 54.7 to − 62.7 ‰ VPDB^8^, fossil methane exhibits a relative enrichment in ^13^C ranging to δ^13^C-CH4 ratios up to − 30‰ VPDB^9,10^. We have traced the fossil methane remaining as dissolved methane in the marine environment and related it to the seasonal oceanographic conditions in the Baltic Sea. Our study aids in estimating the dissolved and released fractions of this highly potent greenhouse gas, resulting from one of the largest known anthropogenic leaks of methane.

## Results and discussion

### Estimations of methane released to the atmosphere by bubble bursting

Immediately after the explosion, a surface bubble plume was identified in pictures from satellites and air reconnaissance^11^. Evidently the methane discharge from the pipeline was strong enough to transport substantial amounts of gas all the way to the sea surface where bubble bursting initiated a direct sea-air gas transfer. Both pipelines Nord Stream (NS) 1 and 2 each consist of two pipes, NS1 A and B as well as NS2 A and B, for a total of four physical pipes. Three of these pipes were affected by detonations, one at three locations^12^. At the time of the explosions, the pipes were not actively transporting gas, but were filled with gas under pressure of approximately 10 MPa. After the explosion at Nord Stream 1, the pressure immediately dropped from 10 to 0.7 MPa at the breach^13^. The extreme event caused a dramatic bubble formation in the water, and the Swedish coastguard reported that the diameter of the bubble plume at the surface was 900 m^14^.

The rate of release to surrounding sea water and emissions to the atmosphere are still under debate. It has been suggested that between 100,000 and 500,000 tonnes of gas were leaked at a rate of 500 tonnes hour^−1^ to the sea during the initial phase of the leakage of approximately one week. The dimensions of the pipeline Nord Stream 1 indicate a possible release of 100,000 metric tonnes from each detonation site during the first 7–8 days. The dimensions of the pipes are approximately the same, and, therefore, we assume that 300,000 metric tonnes were released in total (Fig. 2, phase 1). The European Space Agency satellite GHGSat^11^ observed the plume at the Danish leakage site and estimated an initial emission rate of 79 metric tonnes hour^−1^ to the atmosphere from the Nord Stream 2 breach. If the same release occurred from the three major leaks during the seven days of observed bubbling, it would amount to an emission of 40,000 metric tonnes to atmosphere. A recent study of the load estimated from satellites indicated an emission of 220,000 metric tonnes to the atmosphere^15^.

### Sea-air gas flux when bubble bursting ended

**Figure 1:**
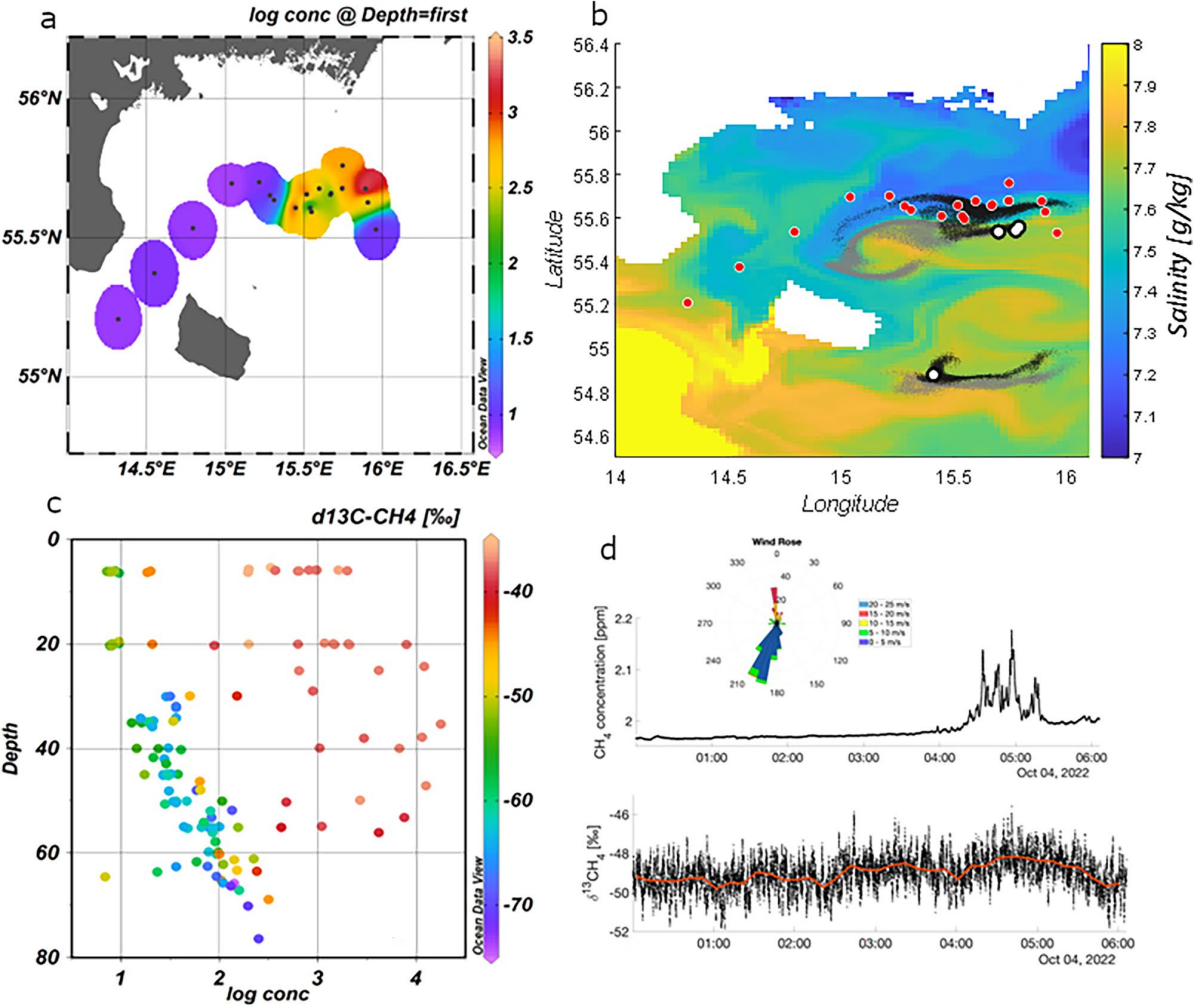
(**a**) Map of the investigation area with near surface methane concentration in colours on a logarithmic scale (log10). Positions for water sampling stations including methane analysis are shown as black dots. (**b**) Model simulation of particles released at the explosion sites. Shown are particles at 6 m (grey) and 36 m (black) on Oct. 3rd, the sites are white dots and red dots are stations. Colour is salinity at 6 m. Current and salinity data is from the Copernicus Marine Data Store (https://doi.org/10.48670/moi-00010). (**c**) Scatter plot of methane concentration versus depth including all the methane data. The colours represent the δ^13^C ratio. Note that data points with high methane concentrations are also enriched in ^13^C corresponding to the δ ^13^C ratio in fossil gas. (**d**) Air concentration of methane showing enhanced values when the ship was located downwind of the explosion sites (see also wind rose). Also shown is the δ^13^C-CH_4_ with higher values during the same event indicating that the source is fossil gas.

**Figure 2:**
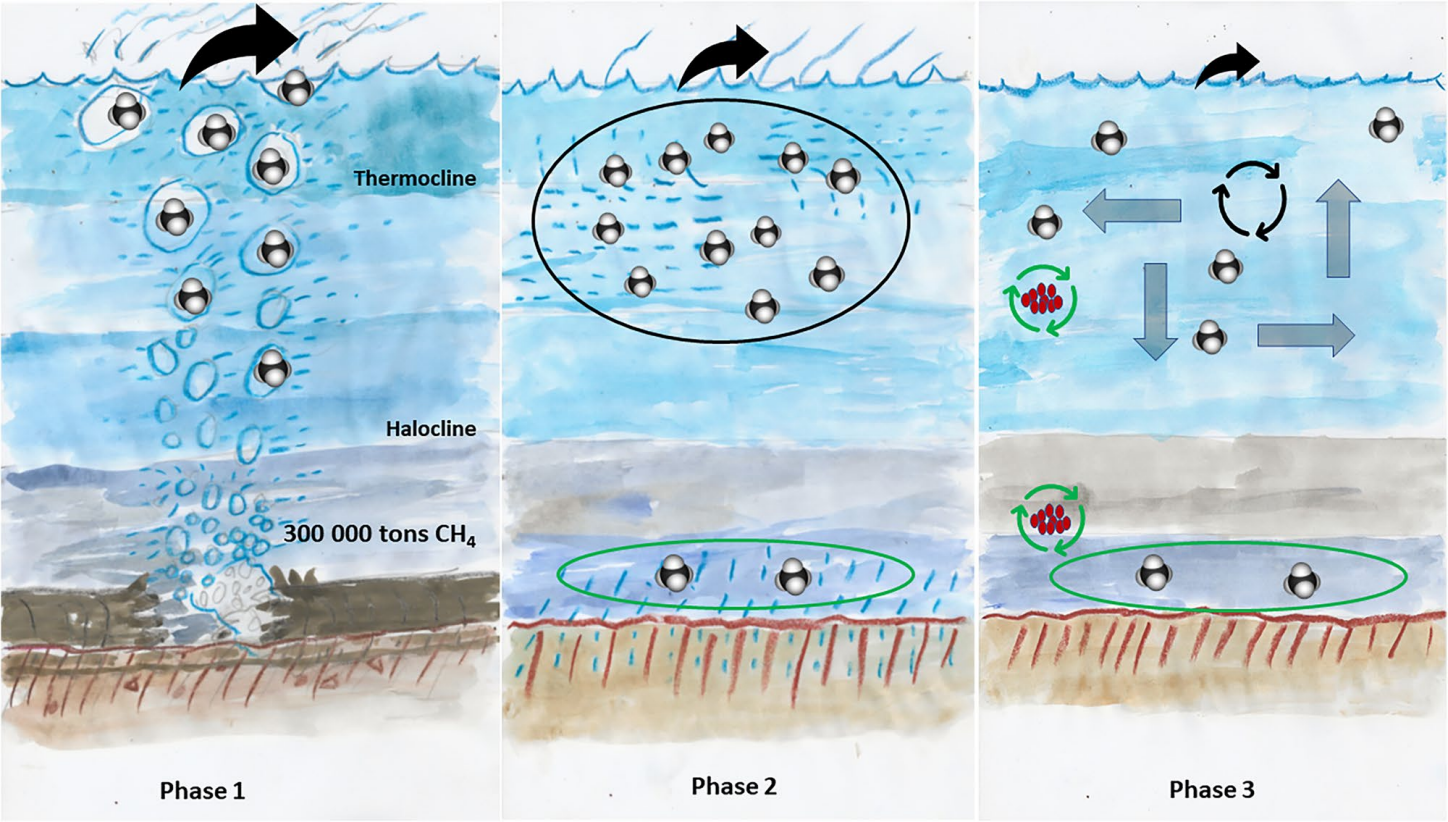
Schematic illustrating three phases of the fate of methane from the explosion at Nord Stream 1 and 2. Phase 1: Immediately after the explosion, 300,000 tonnes of fossil gas was released during the 7 days of bubbling at the surface and the estimated release to the atmosphere was 40,000 tonnes. Phase 2: A week after the event, during our sampling campaign, there was a stable halocline and thermocline. The methane from the pipes were measured and identified in the water column above the halocline. Water above the halocline was super-saturated which could sustain an average flux of air-sea flux of 35 ± 25 nmol m^−2^ s^−1^. Phase 3: Future scenario. During the autumn–winter transition, water will be mixed down to approximately 50 m. The fate we anticipate is a further dilution of the methane from the event which would lead to lower concentration, and thereby a decreasing flux to the atmosphere. Also, an increased methane oxidation rate could be foreseen if methanotrophic bacteria increase in activity and abundance.

While the direct gas transfer to the atmosphere by bubble bursting stopped a week after the detonations, sea-air gas flux initiated by supersaturated surface water still occurred in the second week (Fig. 2, phase 2). Indeed, a shift towards ^13^C-enriched methane in air revealed the direct influence of fossil gas emitted to the atmosphere from the Nord Stream leaks during that time (Fig. 1d). At stations located within the plume area the calculated air-sea fluxes^16,17^ was 100 times higher (35 ± 25 nmol m^−2^ s^−1^; mean ± SD) compared to background stations not affected by fossil methane (0.34 ± 0.21 nmol m^−2^ s^−1^; Fig. 2, phase 2). However, fluxes within the plume area revealed large spatial variations between the stations (e.g., station 10: 8.7 nmol m^−2^ s^−1^, station 14: 90 nmol m^−2^ s^−1^).

### Estimation of methane dissolved in seawater

The phase of bubble bursting, i.e., the direct transfer of fossil gas to the atmosphere, ended after 7 days. However, methane carried in bubbles to the water column which did not reach the sea surface, continued to dissolve at in the water column (Fig. 2, phase 2). To determine the share of fossil methane detected methane in seawater we investigated stations with δ^13^C-CH_4_ values above − 40‰ VPDB with a clear signal of fossil methane. Based on an average methane concentration of 3500 nM and a maximum value of 20,000 nM, the estimation resulted in a total amount between 800 and 4500 metric tonnes of dissolved methane in an area covering 300 km^2^ and water depths down to 50 m. To bring our limited spatial observations into a wider perspective, we performed a model simulation by tracing particles released at all four explosion sites (Fig. 1b). Since the calculation on observations (see above for area and water depth) comprised only 9% of the actual modelled plume we extrapolated the total amount in the water to be about 10,000 to 55,000 metric tonnes.

If we now turn to the estimated total release of 300,000 tonnes, the given values of released methane to the atmosphere during phase 1 of 40,000–220,000 metric tonnes (see above), we note that 27–86% of the total amount released should have been dissolved in sea water. To account for the missing methane, our highest observed concentrations should have been a factor of 1 to 4.7 times higher. We are aware that our estimate inherits a number of uncertainties as it is based on a limited number of measurements. However, we assume that this evaluation underestimates the amount of total dissolved methane, considering that the inner 7 nm circle around the leaks were not sampled.

### Plume formation by methane dissolution in seawater

Apart from transport by bubble bursting and subsequent sea-air flux, the fate of methane dissolved in the water column was considered. We detected a spatial-limited plume, charged with fossil methane (Fig. 1). The model simulation corroborated this distribution pattern showing a primarily westward transport from the northern explosion sites, and an eastward transport from the southern one. Some filaments of particles also went north from the northern sites into our investigation area according to our observations (Fig. 1). The plume shape revealed a lateral and vertical patchy pattern containing up to four orders of magnitude differences in concentration. The pronounced variations in δ^13^C-CH_4_ signatures (Fig. 1) within the plume, compared to the background stations reflect on one side the initial signal from the pipeline (− 37‰VPDB), and on the other side mixing with background methane.

The water stratification evidently influenced the vertical methane spreading. Especially the differences in concentration below and above the halocline, at 50–60 m depth, are remarkable. Bottom water localized below the halocline was free from fossil methane while charged with microbiologically produced methane similar to the background stations further west. This suggests that the power of the explosion was strong enough to transport the gas from the broken Nord Stream pipes immediately above the halocline keeping the bottom water below the halocline almost free from pipeline methane (Fig. 2). By comparison, we detected high variations in concentrations of fossil methane in both subsurface and well-mixed surface water masses each separated by a strong thermocline. As the thermocline is reported to impede transport of dissolved methane to surface water^5^, this pattern indicates bubble transport up the surface water and subsequently dissolution therein, as well as dissolution of methane bubbles in subsurface water on their way upwards (Fig. 2).

## Concluding remarks

To validate the portion of fossil methane which might still be released to the atmosphere during the months following the explosions, water side processes affecting the sink and buffer capacity of the marine environment need to be taken into account (Fig. 2. phase 3). During the transition from autumn to winter, cooling and subsequent convection will break the summer thermocline, which in turn will vertically homogenize the water column down to 50–60 m. Then, the downward transport of the less methane -saturated surface water leads to a dilution of the charge with fossil methane dissolved in the subsurface water. This dilution will affect the sea-air exchange by weakening the concentration gradient at the sea-air interface^18^. In addition, lateral transport of dissolved fossil methane away from the leak sites is likely, as well as within surface waters moving westward towards the Baltic Sea entrance, and within deeper water layers eastwards by frequent inflows of high salinity water from the Kattegat. While mixing and dilution might trigger the buffer capacity, i.e., keeping the fossil methane in the marine environment, microbiological methane oxidation during the upcoming months will act as the final sink. In the water column, methane oxidation naturally occurs by aerobic methanotrophs, a diverse group of microorganisms. Such a methanotrophic bacterial bloom was shown to respire methane following the Deepwater Horizon oil and gas spill in the Gulf of Mexico^19,20^. In fact, the carbon from the oil and methane was recycled in the microbial food web until at least two years later^6^.

Hence, observed high methane concentrations in the surface waters following the Nord Stream gas leak could enable methanotrophic bacteria to increase in abundance and increase methane oxidation activity (Fig. 2, phase 3) and traces could end up in the Baltic Sea microbial food web. Eventually, the ratio of these three processes (dilution, lateral transport, and methane oxidation) to each other will decide the fate of the fossil methane.

## Methods

Our sampling took place on the RV Skagerrak on 3–5 October 2022. In total, 20 stations Northeast of the island Bornholm were covered. 16 sites were located within the methane plume formed by the intense methane bubbling in the week after the explosion, but outside the 7 NM wide security perimeter around the leaks. In addition, we included four sites on the way to the explosion site to measure natural background concentrations (Fig. 1). Profiles of salinity and temperature in the water column were recorded by a Sea-Bird SBE911, a conductivity–temperature–depth (CTD) system. Water samples for the analysis of concentrations of dissolved methane and δ^13^C-CH_4_ values were taken from up to 10 discrete water depths across the entire water with Niskin bottles mounted on a rosette sampler. The deepest bottles were closed at about 1–2 m above the sea floor. The selection of water sampling depths was determined taking the thermocline and halocline depths into consideration.

Sampling procedure was performed as follows: Seawater from the rosette were filled in 500 ml glass vials, sealed with rubber stoppers, and crimped with aluminium caps. Care was taken to avoid any bubbles. A headspace was created by injecting 20 ml hydrocarbon free synthetic air while at the same time water was pushed out via a second needle to balance the pressure. When equilibrated, a subsample was removed with a gas tight syringe and injected to the Small Sample Isotope Module (SSIM) coupled to a Picarro G2132-i cavity ring-down spectrometer. For calibration, we used standard gas mixtures with different concentrations (Fa. *Linde*), and standard gas mixtures with isotopic ratios of − 25‰ vs. VPDB, − 45‰ vs. VPDB and 69‰ vs. VPDB (Fa Airgas). Considering the complete sampling procedure, the overall total uncertainty was < 5% estimated on duplicates. The calculation of the dissolved methane concentration followed Wiesenburg and Guinasso^17^.

During the night, atmospheric methane concentration and δ^13^C-CH_4_ was measured continuously. The time series included the periods from 3 October 19:50 to 4 October 05:55, from 4 October 22:10–to 5 October 06:00, from 5 October 19:30–to 6 October 15:00. Air was continuously drawn from the forward mast of the R/V Skagerak at about 12 m above water surface using a Teflon tube inserted in the Picarro G2132-i. The Picarro G2132-i continuously measured at high precision mode (HP mode with 3 Hz). A constant flow rate was generated with a Boxer 3KQ diaphragm pump.

The sea-air CH_4_ flux (*F*) was determined with the flux equation [Eq. (1)], following Wanninkhof and colleagues^16^, assessing the gradient between the measured surface concentration of CH_4_ (*C*_*w*_) and the concentration of CH_4_ in equilibrium with the atmosphere (*C*_*a*_) calculated with the atmospheric CH_4_ measurements averaged for the

1 $$F \, = \, k \, (C_{w} - C_{a} )$$

2 $$k \, = \, \left( {0.251 \, u_{10}^{2} \left( {Sc/660} \right)^{ - 0.5} } \right)$$

time at each station. The gas transfer velocity (*k*) was determined using the Schmidt number (Sc), which is calculated as a function of water temperature [Eq. (2)]^17^. The *in-situ* ship-based measurements of the wind speed during the corresponding time at each station were corrected to 10 m height, following Amorocho and DeVries^21^. Positive *F* values indicate gas emissions from the surface water to the atmosphere, while negative values of *F* indicate an uptake (or sink) of the gas in the surface water.

Simulation of Lagrangian particles released at the explosion sites were performed using hourly currents from the Copernicus Marine Data Store (MDS) derived Baltic Sea Physics Reanalysis^22^ dataset. In the first two days after the explosion 50% of particles were released, and the remaining particles over the next 6 days (in total 292,000 particles at each depth). The particles were tracked until October 6, 15:00 using a matlab code that applies built in functions for linear interpolation of velocities to particle positions and a first order advection scheme is applied to advect the particles in the velocity field. The particles are seeded around the release position using a random distribution, and we also use a small random advection speed (mean 0.0125 m s^−1^) to mimic horizontal turbulence. We have tracked particles horizontally at each depth of the hydrodynamical model, and evaluated the number of particles that are found in the target area defining the observational stations. The depth of the methane dissolution, and consequent interlayering of water from the release sites, is not well known and the same number of particles were therefore released at each depth. Most particles found in the target area are between 30 and 50 m, in accordance with observations. Due to these uncertainties, we used the results from depths 10, 28, and 54 m. The total number of model particles at these depths found in the observational area is 10, 9, and 8% at noon of the 4th, 5th, and 6th of September respectively, and from this we evaluated that roughly 9% of particles in the simulation would be observable by the field campaign.

## Data Availability

The datasets generated and/or analysed during the current study will be available in the PANGAEA repository (www.pangaea.de). The data will be given a DOI number after publication. The data from the current study is available from the corresponding authors on reasonable request.
